# Disrupted Structural Brain Connectome Is Related to Cognitive Impairment in Patients With Ischemic Leukoaraiosis

**DOI:** 10.3389/fnhum.2021.654750

**Published:** 2021-06-10

**Authors:** Tong Lu, Zan Wang, Ying Cui, Jiaying Zhou, Yuancheng Wang, Shenghong Ju

**Affiliations:** ^1^Nanjing Medical University, Nanjing, China; ^2^Department of Radiology, Zhongda Hospital, School of Medicine, Southeast University, Nanjing, China; ^3^Department of Neurology, Zhongda Hospital, School of Medicine, Southeast University, Nanjing, China

**Keywords:** ischemic leukoaraiosis, diffusion tensor imaging, white matter integrity, graph theory, structural brain network, brain connectome, cognitive impairment

## Abstract

Ischemic leukoaraiosis (ILA) is related to cognitive impairment and vascular dementia in the elderly. One possible mechanism could be the disruption of white matter (WM) tracts and network function that connect distributed brain regions involved in cognition. The purpose of this study was to investigate the relationship between structural connectome and cognitive functions in ILA patients. A total of 89 patients with ILA (Fazekas score ≥ 3) and 90 healthy controls (HCs) underwent comprehensive neuropsychological examinations and diffusion tensor imaging scans. The tract-based spatial statistics approach was employed to investigate the WM integrity. Graph theoretical analysis was further applied to construct the topological architecture of the structural connectome in ILA patients. Partial correlation analysis was used to investigate the relationships between network measures and cognitive performances in the ILA group. Compared with HCs, the ILA patients showed widespread WM integrity disruptions. The ILA group displayed increased characteristic path length (*L*_p_) and decreased global network efficiency at the level of the whole brain relative to HCs, and reduced nodal efficiencies, predominantly in the frontal–subcortical and limbic system regions. Furthermore, these structural connectomic alterations were associated with cognitive impairment in ILA patients. The association between WM changes (i.e., fractional anisotropy and mean diffusivity measures) and cognitive function was mediated by the structural connectivity measures (i.e., local network efficiency and *L*_p_). In conclusion, cognitive impairment in ILA patients is related to microstructural disruption of multiple WM fibers and topological disorganization of structural networks, which have implications in understanding the relationship between ILA and the possible attendant cognitive impairment.

## Introduction

Ischemic leukoaraiosis (ILA), also referred to as white matter hyperintensities (WMHs) or age-related white matter (WM) changes, is characterized by bilateral, patchy, or diffuse areas of hyperintensities with different severity on T_2_-weighted or fluid-attenuated inversion recovery (FLAIR) sequence in the elderly (Wardlaw et al., 2013; Prins and Scheltens, 2015; Alber et al., 2019). Convergence evidence suggested that WMHs are involved in cognitive impairment of various populations, including normal-aging, vascular dementia, Type 2 diabetes mellitus, and Alzheimer’s Disease (AD; Debette and Markus, 2010; Zhang et al., 2014). Previous studies have shown that normal elderly people with ILA are at the risk of further cognitive deterioration, and the burden of WMHs is positively correlated with the severity of cognitive decline (Te et al., 2015; Chen et al., 2016; Zeestraten et al., 2017; Zeng et al., 2020). However, the effects of ILA on cognitive abilities are insidious and can be difficult to detect at an early stage but are nevertheless crucial.

Diffusion tensor imaging (DTI) has been developed as a powerful noninvasive technique to investigate WM microstructural integrity since it is sensitive to the microstructural damage of both the normal appearing WM and WMHs (Le Bihan et al., 2001; Papma et al., 2014; Tuladhar et al., 2017; Tae et al., 2018; Mascalchi et al., 2019). Increasing evidences have confirmed that cognitive function is strongly associated with WM integrity detected by DTI (Li et al., 2012; Chen et al., 2020). As reported previously based on the tract-based spatial statistics (TBSS) technique, the atrophy and reduced diffusion anisotropy of the corpus callosum (CC) may indicate diffuse deep WM destruction in ILA, which may explain global cognitive decline and progression of vascular dementia (Otsuka et al., 2012). Many studies have revealed that fractional anisotropy (FA) and mean diffusivity (MD) are sensitive indices for use in evaluating their relationships with cognitive impairment in ILA patients (Della Nave et al., 2007). However, few studies have explored the association of the topological characteristics and network properties of the whole-brain WM connectome with cognitive impairment in patients with ILA.

Nowadays, graph theory analysis has been increasingly applied to construct a WM structural network and explore structural network topological organization in cerebral small vessel disease (SVD) (Tuladhar et al., 2015; Yang et al., 2020). However, an important question is the inconsistency among previous studies. Given patients with lacunar stroke and leukoaraiosis (i.e., ILA) have a relatively homogeneous pattern of cognitive impairment (Román et al., 2002), it is important to recruit ILA patients to investigate the effect of SVD on WM microstructural integrity and topological organization of structural brain connectome. Therefore, in this relatively homogeneous study, we conducted a relatively larger-sample, case-control study to investigate the WM integrity and structural network topological characteristics present in patients with ILA by TBSS and graph theoretical approaches. We further tried to elucidate whether the structural connectomic disruptions could explain cognitive dysfunction in ILA patients.

## Materials and Methods

### Study Participants

Initially, we recruited 179 elder, naturally right-handed Han Chinese individuals, including 89 consecutive subjects with moderate to severe WMHs (defined as a sum of the deep WMH Fazekas score and the periventricular WMH Fazekas score ≥ 3 on FLAIR sequence images) and 90 healthy controls (HCs) without moderate to severe WMHs (Fazekas score = 0) and lacunes. Two ILA patients and three HCs were excluded because of head motion or incomplete image coverage. The remaining 87 ILA patients and 87 HCs were left for further analyses.

The study was approved by the Research Ethics Committee of Zhongda Hospital Affiliated to Southeast University. Written informed consent was obtained from all participants before inclusion. The details on the inclusion and exclusion criteria can be found in Supplementary Material.

### Cognitive Assessment

We assessed general cognitive performance for all participants using the mini-mental state examination (MMSE), and performed a concise neuropsychological test battery to evaluate multiple cognitive domains, such as episodic memory, visuospatial ability, information processing speed, and executive function. This battery consisted of auditory verbal learning test (AVLT) and its 20-min delayed recall (AVLT-DR), Rey–Osterrieth complex figure test (CFT) and its 20-min delayed recall (CFT-DR), logical memory test (LMT) and its 20-min delayed recall (LMT-DR), clock drawing test (CDT), digital span test (DST), digital symbol substitution test (DSST), verbal fluency test (VFT), trail-making test-A (TMT-A), trail-making test-B (TMT-B), Stroop color-word test (Stroop), and semantic similarity test (Similarity).

### MRI Scans

Magnetic resonance imaging (MRI) was performed with a clinical 3-T scanner (Siemens Healthcare, Erlangen, Germany) using a 12-element head coil. A high-resolution T_1_-weighted three-dimensional magnetization prepared rapid acquisition gradient echo sequence covering the whole brain was applied for anatomic reference with the following parameters: repetition time (TR) = 1,900 ms, echo time (TE) = 2.48 ms, flip angle = 9°, matrix size = 256 × 256, field of view (FOV) = 250 mm × 250 mm, slice thickness = 1.0 mm, gap = 0 mm, and 176 slices. Diffusion-weighted MRI covering the whole brain consisted of a diffusion-sensitized echo-planar imaging sequence [TR = 10,000 ms, TE = 90 ms, flip angle = 90°, 70 slices, slice thickness = 2 mm without interslice gap, matrix size = 128 × 128, FOV = 256 mm × 256 mm, number of excitation (NEX) = 2.0, 30 diffusion sensitizing gradient directions (*b* = 1,000 s/mm^2^)] with one single image with no diffusion weighting (*b* = 0 s/mm^2^). Additionally, axial T_2_-weighted, diffusion-weighted imaging sequence, and susceptibility-weighted imaging were performed to detect acute or subacute infarctions and cerebral microbleeds.

### Image Preprocessing and Diffusion Tensor Tractography

The DTI data preprocessing and analyses were carried out using the FSL toolbox^1^ and the PANDA software.^2^ The preprocessing of DTI data consisted of the following steps: correcting for the eddy current distortions and head motion artifacts by applying the diffusion-weighted images to the b0 images with an affine transformation, estimating the diffusion tensor, and calculating the FA and MD. The voxel-wise statistical analysis of FA and MD data was carried out using TBSS, part of the FSL tools. Automated atlas-guided WM tract reconstruction was performed to determine the spatial distribution of WM tracts. Diffusion-weighted MRI analysis comprised whole-brain WM tractography as described previously (Wang et al., 2020b; Supplementary Table 1).

### WM Network Construction

To construct the structural brain network, network nodes were first defined as 90 cortical and subcortical regions (45 for each hemisphere, Supplementary Table 2) segmented by the automated anatomical labeling (AAL) template (Tzourio-Mazoyer et al., 2002). For each pair of brain regions/nodes defined above, fibers with two endpoints located in their respective masks were considered as the network edges structurally connecting the two nodes. We identified the fiber number (FN) of the connected WM fibers between two regions as the weights of the network edges and constructed the FN-weighted WM network, representing by a 90 × 90 connectivity matrix for each subject. Detailed descriptions of the network construction are provided in the Supplementary Material.

### Network Topology Analyses

To further characterize the structural brain connectome, we performed a whole-brain network analysis using the Gretna Toolbox (Wang et al., 2015). We computed global network parameters, including (1) small-world properties (Watts and Strogatz, 1998) involving characteristic path length (*L*_p_), clustering coefficient (*C*_p_), normalized characteristic path length (*λ*), normalized clustering coefficient (*γ*), and small-worldness (*σ*); and (2) network efficiency involving global efficiency (*E*_glob_) and local efficiency (*E*_loc_). Furthermore, we calculated the nodal efficiency (*E*_nodal_) to identify the regional (or nodal) characteristics of the brain networks. The graph theoretical analysis employed to obtain these network measures was previously detailed (Rubinov and Sporns, 2010; Bai et al., 2012).

### Statistical Analysis

#### Demographic and Neuropsychological Data

The two-independent-sample *t*-test and chi-square test were used to test the between-group differences in the demographic data. One-way analyses of covariance (ANCOVAs) were conducted to explore the group differences in neuropsychological performances, with age, sex, and education years as covariates. *P* < 0.05 was considered to indicate a statistically significant difference. All analyses were performed with the SPSS version 22.0 software (SPSS, Inc., Chicago, IL, United States).

#### WM Tract Integrity

After correcting for age, sex, and education level, tract-specific ANCOVAs were employed to investigate the group differences in FA and MD values on each WM tract region of interest (ROI). Multiple comparisons were corrected by false discovery rate (FDR) method. *P*-values < 0.05 (FDR-corrected) were considered statistically significant.

#### Network Topological Metrics

Between-group differences in topological attributes at the level of network were investigated by nonparametric permutation tests (Bai et al., 2012; Wang et al., 2016; Supplementary Material). To be noted, the effects of age, sex, and education level were removed for each network metric by a regression analysis performed before the network analysis of structural connections.

#### Association Between Network Measures and Cognition Function

To further identify the clinical relevance of the altered structural connectivity in ILA patients, we correlated the cognitive function with network topologies. Partial correlation analyses were conducted in ILA group adjusting for age, sex, and educational level. A *P*-value < 0.05 was considered statistically significant.

#### Mediation Analysis

Mediation analysis was performed to investigate whether alterations in structural connectivity were involved in the relationship between MRI measures of diffuse WM damage and cognitive function, adjusting for age, sex, and education level. We used bootstrapping (10,000 samples) to calculate bias-corrected 95% confidence intervals for the size of the mediating effects with the PROCESS statistical package for the SPSS 22.0 framework.

## Results

### Demographic and Neuropsychological Results

Demographic data and cognitive performances for the ILA and control groups are summarized in Table 1. No significant differences were found in sex or years of education between ILA and control subjects. Notably, the age effect was removed in all of the following cognitive, WM microstructural integrity, and network analyses. In the ILA group, cognitive function in cognitive domain episodic memory, information processing speed, and executive function were significantly worse than HCs. However, no significant group differences were observed in the cognitive domain visuospatial function.

**TABLE 1:** Demographic and neuropsychological data for ILA and control groups.

	**HCs (*n* = 90)**	**ILA (*n* = 89)**	***T*/χ^2^**	***P*-value**
**Demographic data**				
Age (years)	65.6 ± 6.8	72.6 ± 6.9	–6.791	< 0.001^a^
Sex (male/female)	35/55	42/47	1.258	0.262^b^
Education (years)	12.3 ± 2.8	11.5 ± 3.4	1.579	0.116^a^

**Neuropsychological test data**			***F***	***P*-value**

MMSE	28.29 ± 1.41	27.02 ± 2.96	1.004	0.318^c^
Composite Z scores of each cognitive domain				
Episodic Memory	0.50 ± 0.52	−0.50 ± 0.79	51.650	0.000^c^
AVLT-DR (raw score)	7.42 ± 2.20	3.91 ± 2.97	34.705	0.000^c^
LMT-DR (raw score)	8.14 ± 2.53	4.68 ± 3.42	36.430	0.000^c^
CFT-DR (raw score)	19.44 ± 5.92	12.34 ± 7.98	19.399	0.000^c^
Visuospatial Function	0.17 ± 0.59	−0.17 ± 0.86	0.342	0.559^c^
CFT (raw score)	32.44 ± 5.34	31.29 ± 6.36	0.118	0.731^c^
CDT (raw score)	8.81 ± 1.23	8.04 ± 1.77	1.530	0.218^c^
Processing Speed	0.37 ± 0.66	−0.37 ± 0.87	6.555	0.011^c^
DSST (raw score)	42.03 ± 11.61	29.47 ± 12.43	9.411	0.003^c^
TMT-A (raw score, second)	61.91 ± 16.50	87.46 ± 37.95	8.983	0.003^c^
Stroop A (raw score, second)	26.58 ± 4.77	32.32 ± 9.56	4.384	0.038^c^
Stroop B (raw score, second)	40.23 ± 11.55	48.94 ± 14.97	2.701	0.102^c^
Executive Function	0.33 ± 0.54	−0.33 ± 0.74	13.588	0.000^c^
VFT-objects (raw score)	25.23 ± 6.13	18.92 ± 6.46	23.433	0.000^c^
VFT-animals (raw score)	20.32 ± 5.18	16.71 ± 5.69	7.122	0.008^c^
DST-backward (raw score)	4.99 ± 1.47	4.18 ± 1.58	2.058	0.153^c^
TMT-B (raw score, second)	159.68 ± 46.07	227.25 ± 116.83	3.520	0.062^c^
Stroop C (raw score, second)	80.62 ± 22.99	102.09 ± 41.64	2.963	0.087^c^
Similarity (raw score)	19.14 ± 3.07	15.93 ± 4.48	16.648	0.000^c^

*All of the subjects were matched for sex and education. Data are presented as the means ± SD. HCs, healthy controls; ILA, ischemic leukoaraiosis; MMSE, mini-mental state examination; AVLT-DR, auditory verbal learning test-20 min delayed recall; LMT-DR, logical memory test-20 min delayed recall; CFT-DR, Rey–Osterrieth complex figure test-20 min delayed recall; CFT, Rey–Osterrieth complex figure test; CDT, clock drawing test; DSST, digital symbol substitution test; TMT-A, trail making test-A; Stroop, stroop color and word tests; VFT, verbal fluency test; DST, digit span test; TMT-B, trail making test-B; Similarity, semantic similarity test. ^*a*^Independent-sample *t*-test. ^*b*^*χ*^2^ test. ^*c*^Analysis of covariance (ANCOVA).*

### WM Tract Integrity

**FIGURE 1:**
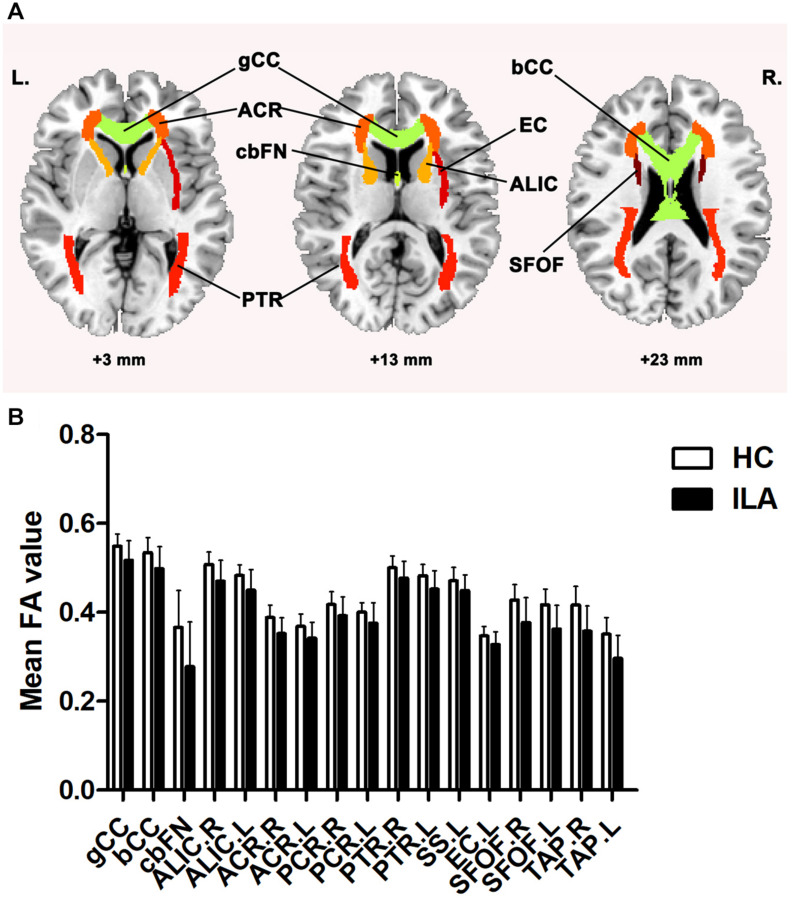
**(A)** ILA-related disruption in WM tracts. **(B)** Compared with HCs, the ILA group displayed significantly lower FA in widespread atlas-based tract ROIs after FDR correction. For the abbreviations of WM tracts, see Supplementary Table 1. HC, healthy controls; ILA, ischemic leukoaraiosis; FA, fractional anisotropy. L, left; R, right.

Figure 1 displays the mean FA and MD measures of each tract ROI with significant between-group differences after FDR correction in the ILA and HC groups. Compared with the HCs, the ILA patients exhibited significantly lower FA values in the genu of corpus callosum (gCC), body of corpus callosum (bCC), fornix column and body of fornix (cbFN), left sagittal stratum (SS.L), left external capsule (EC.L), bilateral anterior limb of internal capsule (ALIC), anterior corona radiata (ACR), posterior corona radiata (PCR), posterior thalamic radiation (PTR), superior fronto-occipital fasciculus (SFOF), and tapetum (*P* < 0.05, FDR-corrected). However, there were no significant tract-specific MD differences between the two groups at *P* < 0.05 after FDR correction.

### Small-World Properties of the WM Structural Networks

#### ILA-Related Alterations in Global Topology

Statistical analyses showed significant differences in both small-world parameters and network efficiency between the ILA patients and HCs (Supplementary Table 3). Permutation tests revealed significantly increased characteristic *L*_p_ (*P* < 0.001) and normalized *C*_p_ (*P* = 0.0011) in the ILA patients relative to the HCs. As for topological efficiency, the WM structural networks of ILA patients demonstrated decreased global efficiency *E*_glob_ (*P* < 0.001) compared with those of HC subjects. We also evaluated the effects of different thresholds on the network analysis by setting threshold values of the number of fiber bundles ranging from 1 to 5. We found that the threshold procedure did not significantly influence our results (Supplementary Figure 2).

#### ILA-Related Alterations in Regional Efficiency

**FIGURE 2:**
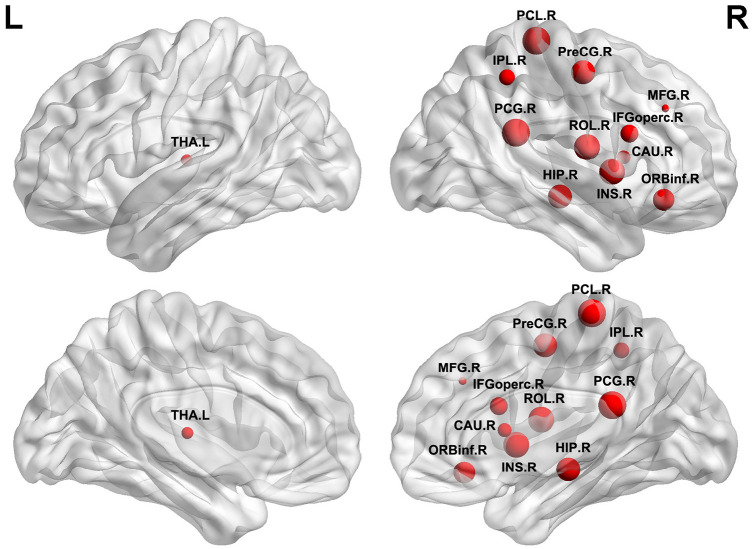
Brain regions identified as abnormal nodal efficiency in ILA. Red nodes show the regions of reduced efficiency in patients with ILA relative to healthy controls. The node sizes denote the significance of between-group differences in the regional efficiency. For the abbreviations of nodes, see Supplementary Table 2. L, **left**; R, **right**.

To further explore the effect of this disorganization on nodal characteristics of the WM networks, we investigated the group differences in nodal efficiency. Compared with HCs, the ILA patients displayed a widespread reduction in nodal efficiency in many frontal–subcortical and limbic system regions [permutation with 10,000 tests, corrected for *N* = 90 multiple comparisons with false positive correction *P* < (1/*N*) = 0.011], including four frontal regions [right precentral gyrus (PreCG.R), right middle frontal gyrus (MFG.R), right opercular part of inferior frontal gyrus (IFGoperc.R), and right orbital part of inferior frontal gyrus (ORBinf.R)], right rolandic operculum (ROL.R), right insula (INS.R), right posterior cingulate gyrus (PCG.R), right hippocampus (HIP.R), right inferior parietal (IPL.R), right paracentral lobule (PCL.R), and two subcortical regions [left thalamus (THA.L) and right caudate nucleus] (Figure 2).

#### Association Between WM Network Measures and Cognition in ILA Patients

**FIGURE 3:**
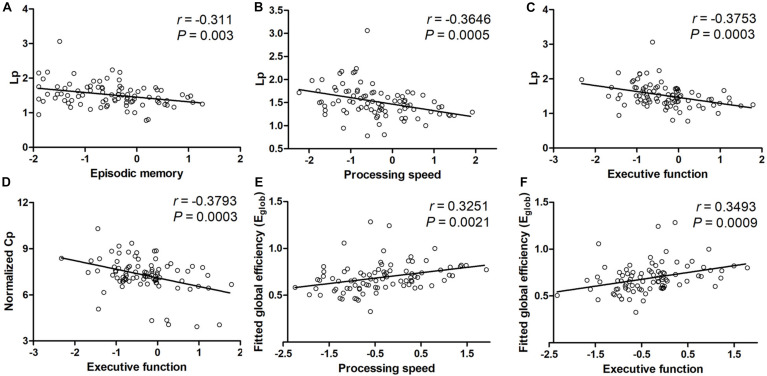
The relationships of WM network topological properties and neuropsychological performances in patients with ILA. **(A–C)** Within the ILA group, the characteristic path length *L*_p_ exhibited significant correlations with three cognitive domains [episodic memory (*Z* scores): *r* = −0.311, *P* = 0.003; processing speed (*Z* scores): *r* = −0.3646, *P* = 0.0005; executive function (*Z* scores): *r* = −0.3753, *P* = 0.0003]. **(D)** The normalized *C*_p_ correlated negatively with executive function (*Z* scores; *r* = −0.3793, *P* = 0.0003) in ILA patients. **(E,F)** The global efficiency *E*_glob_ correlated positively with the cognitive domains of processing speed (*Z* scores; *r* = 0.3251, *P* = 0.0021) and executive function (*Z* scores; *r* = 0.3493, *P* = 0.0009) in ILA patients. ILA, ischemic leukoaraiosis.

We further examined the relationships between WM network topological properties and neuropsychological scores in the patients with ILA (Figure 3). Within the ILA group, the characteristic path length *L*_p_ correlated negatively with the cognitive domains of episodic memory (*r* = −0.311, *P* = 0.003), information processing speed (*r* = −0.3646, *P* = 0.0005), and executive function (*r* = −0.3753, *P* = 0.0003). However, no significant associations between network topological measures and domain-specific cognitive performances were observed in the HC group.

**FIGURE 4:**
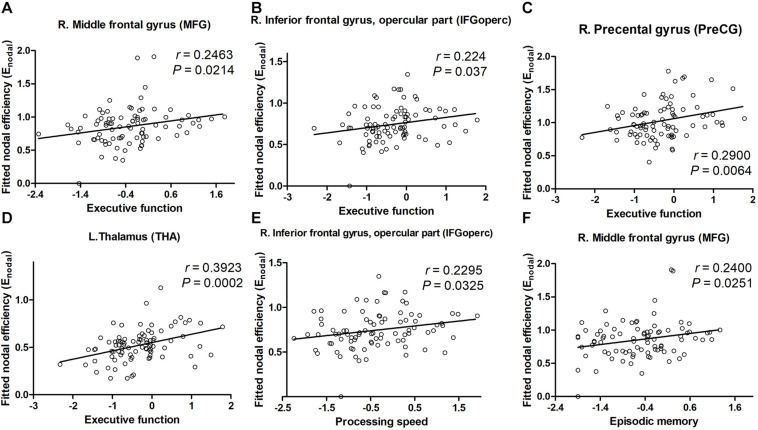
The significant correlations between cognitive performances and network parameters in patients with ILA. **(A–D)** Within the ILA group, the executive function (*Z* scores) correlated positively with the nodal efficiency of the MFG.R, IFGoperc.R, PreCG.R, and THA.L. **(E,F)** The nodal efficiency of the MFG.R correlated significantly with the episodic memory (*Z* scores) in patients with ILA, of the IFGoperc.R with the processing speed (*Z* scores). For the abbreviations of nodes, see Supplementary Table 2. ILA, ischemic leukoaraiosis.

For nodal characteristics, we investigated only the nodes with significant group differences. The executive function correlated positively with the nodal efficiency of the PreCG.R, MFG.R, IFGoperc.R, ROL.R, IPL.R, and THA.L (all *P*s < 0.05, Figure 4) in the ILA group. Nodal efficiency of the IFGoperc.R, ROL.R, IPL.R, and THA.L correlated with processing speed (all *P*s < 0.05). In addition, the cognitive domain episodic memory correlated positively with the nodal efficiency of the MFG.R, ROL.R, and THA.L (all *P*s < 0.05).

#### Mediation Path Between WM Structure Measures and Cognition in ILA Patients

**FIGURE 5:**
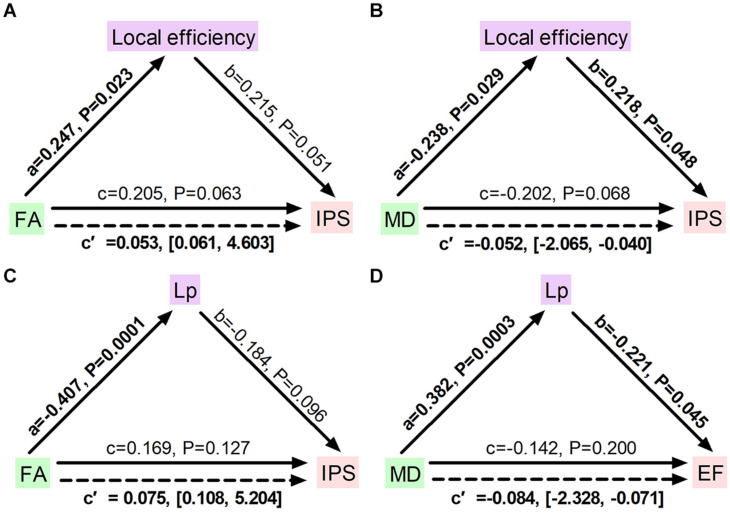
Diagrams illustrating statistical mediation of the association between FA and MD measures and cognition by network properties in ILA. **(A)** Association between FA and information processing speed was significantly mediated by local efficiency. **(B)** Association between MD and information processing speed was significantly mediated by local efficiency. **(C)** Association between FA and information processing speed was significantly mediated by characteristic path length. **(D)** Association between MD and executive function was significantly mediated by characteristic path length. For each connection, the standard regression coefficient (standardized *β*) and 95% confidence interval of the indirect mediation effect (c’) were shown. Solid lines represent direct effects (c); dashed lines represent indirect effects (c’). Significant pathways appear in bold. *L*_p_, characteristic path length; FA, fractional anisotropy; MD, mean diffusivity; IPS, information processing speed; EF, executive function.

To further identify whether disrupted network topology could fully or partially bridge WM integrity and cognitive impairment, mediation models were constructed among altered network measures, WM diffusion metrics, and cognition in ILA patients. We found that local efficiency significantly mediated the association between FA measures and the information processing speed (indirect effect: 0.053; 95% confidence interval: 0.061, 4.603; Figure 5A) and the association between MD and the information processing speed (indirect effect: −0.052; 95% confidence interval: −2.065, −0.040; Figure 5B). Additionally, characteristic path length significantly mediated the association between FA measures and the information processing speed (indirect effect: 0.075; 95% confidence interval: 0.108, 5.204; Figure 5C) and the association between MD and the executive function (indirect effect: −0.084; 95% confidence interval: −2.328, −0.071; Figure 5D). Aside from significant mediations on local efficiency and characteristic path length, no other significant mediations were observed in the ILA group.

## Discussion

Using DTI tractography and network analysis, we demonstrated the disrupted WM integrity and topological alterations of WM networks in ILA. Our main results are as follows: (1) WM tract integrity was extensively disrupted in ILA patients as indicated by significantly reduced FA in several fiber bundles including the CC, EC.L, bilateral ALIC, ACR, PCR, PTR, SFOF, and tapetum; (2) the global topological organization of WM structural networks in ILA patients was significantly disrupted as indicated by abnormal small-world properties and topological efficiency; (3) the regional characteristics (i.e., nodal efficiency) were reduced predominantly in the frontal–subcortical and limbic system areas in ILA patients; (4) these structural connectomic alterations correlated with the cognitive performances in the ILA group; and (5) the relationship between WM abnormalities (i.e., FA and MD measures) and domain-specific cognitive function was mediated by the structural connectivity measures (i.e., local network efficiency and characteristic path length). Altogether, the network-level alterations identified in this study provide strong support that the core aspects of the pathophysiology of this disease are associated with disruption of the large-scale brain networks and enhance our understanding of the neuropathological mechanisms underlying the ILA-related cognitive impairment.

A growing number of studies have applied DTI to explore WM integrity and the role of specific WM tracts in the process of ILA-related cognitive decline. In agreement with previous studies in WMH patients (Tuladhar et al., 2015; D’Souza et al., 2018), we found widespread abnormalities of many specific WM tracts in ILA patients, including the CC, EC.L, bilateral ALIC, ACR, PCR, PTR, and SFOF. Since these fiber tracts form the important anatomical connectivity or circuits, they may be directly relevant to the pathophysiology of ILA. To date, there are several studies suggesting various WM impairments in ILA patients. A recent TBSS study reported that the gCC exhibited the most damaged WM fiber in patients with subcortical ischemic vascular disease and some specific WM tracts were significantly correlated with the severity of WMHs, cognitive assessments about executive functions, and processing speed (Liu et al., 2019). Considered to be the most important imaging manifestations reflecting the overall burden of SVD, WMHs may disturb WM fibers connected to the cortical to subcortical gray matter, leading to secondary damage of axonal cytoskeleton and cortical degeneration (Jones et al., 1999; Du et al., 2005). Another study also showed that DTI may provide more important information about the ILA-related cognitive impairment, which might be possibly attributed to a disconnection syndrome of cortico-subcortical pathways (Yuan et al., 2017). Our results added to the growing body of literature that implicated specific disruption of WM tracts (i.e., CC, thalamic radiation) connected between cortical and subcortical regions in ILA (Glickstein and Berlucchi, 2008; Duering et al., 2011), which may cause characteristic structural and functional alterations of frontal–subcortical network and lead to cognitive impairment eventually. The microstructural integrity in the genu and splenium showed the highest significant relation with global cognitive function and executive functions, in the cingulum bundle with verbal memory performance (Tuladhar et al., 2015). A recent multi-modality MRI study has shown that WMHs, combined with the disruption of passing-through fiber integrity and altered functional activities in areas connected by this fiber, are associated with a decline of psychomotor processing speed (Wang et al., 2020a). Moreover, the widespread WM microstructural impairments disrupt the large-scale distributed brain cognitive networks and underlie the various cognitive dysfunctions in ILA. Although these findings were observed at both the regional and the voxel-level, further investigations should be performed to verify the relationship and explore the mechanisms.

Graph theoretical analysis is applied to study the whole brain connectivity and provide information on the amount of integrations among brain regions. Despite having a common small-worlded topology similar to HCs in general terms, the ILA patients demonstrated significantly disrupted measures of structural network properties. The ILA patients showed an increased absolute path length in their brain networks as compared with HCs. Furthermore, network efficiency analyses revealed abnormal small-world organization in ILA group, as characterized by reduced global efficiency. Our results are compatible with several existing connectome-based studies on SVD (Lawrence et al., 2014; Tuladhar et al., 2016, 2017; Du et al., 2019). Given that the small-world model reflects an optimal balance between local specialization and global integration (Bullmore and Sporns, 2012), these findings suggest a disturbance of the normal balance in the structural networks in ILA. Therefore, the ILA-related alterations in the absolute path lengths and global network efficiency might be attributed to underlying disconnections between affected brain regions.

Intriguingly, our results showed that the topological alterations (i.e., longer path length and impaired global network efficiency) significantly correlated with cognitive deficits in patients with ILA. We also found that the local but not the global network efficiency significantly mediated the relationship between FA and MD measures and the cognitive domain information processing speed. Meanwhile, characteristic path length acted as a mediator between FA and MD measures and cognitive function. These findings highlight the importance of network impairment as a mediating framework between WM changes and cognitive decrements in ILA. Previous studies on SVD have demonstrated that alterations in the structural network organization (i.e., reduced global and local efficiency) are associated with the MRI markers for SVD (i.e., WMHs, cerebral lacunar infarcts, and microbleeds), and topological disorganization mediates the relationship between MRI markers and cognition in SVD (Lawrence et al., 2014; Tuladhar et al., 2016). Furthermore, a recent clinical study suggested that WMH volumes, structural connectivity measures (i.e., local network efficiency), and information processing speed were interrelated, and the relationship between WMHs and information processing speed was mediated by the local network efficiency (Vergoossen et al., 2021). In addition, emerging literatures on SVD reported that network efficiency could mediate the associations between cerebral vascular lesions and cognitive function, which shed light on the importance of connectome-based analyses in understanding the precise underlying topological mechanism of cognitive decrements in SVD (Du et al., 2019). Our present study is compatible with previous literature and supports the idea that the network metrics have potential as markers for SVD-related cognitive dysfunction. An interaction analysis of regional gray matter and WMH volume, network connectivity, WM integrity, and cognitive impairment is worthy of further investigation in our future study.

Next, we investigated global network efficiency at a nodal level to know the extent of information transmission capacity of nodes with all other nodes in brain networks. In this study, 12 brain nodes showed decreased nodal efficiency in patients with ILA. To briefly summarize, most of the altered nodes were generally located in the frontal–subcortical (PreCG.R, MFG.R, IFGoperc.R, and ORBinf.R) and limbic system (INS.R, HIP.R, PCG.R, and THA.L) in terms of spatial anatomic location, which matched with previously structural studies showing that network connectivity was altered between WMHs and cognitive impairment in SVD (Lawrence et al., 2014). These regional abnormalities may cause a segregation of different brain systems and yield a disruptive integration of large-scale brain networks. Specifically, structural MRI studies showed that WMHs can disrupt the integrity of WM fibers and damage structural connections (Reginold et al., 2018; Chen et al., 2020), resulting in disrupted topological properties of nodes connected by the fibers. Evidences are also supported by functional MRI results showing that the network efficiency is significantly correlated with the level of cognitive impairment in patients with WM lesions (Chen et al., 2019; Wang et al., 2019). Nevertheless, it is interesting that we found that there were more abnormal nodal characteristics in the right hemisphere than in the left. The mechanisms of how ILA affects hemispheric symmetry of nodal network properties are worthy of further study. Our results exhibit widespread sporadic disruption of WM structural networks in most regions, leading to obvious network disorders that are important for cognitive processes in ILA.

In this present study, we further evaluated the relationships between WM network metrics and cognition in patients with ILA. Importantly, nodal efficiencies of the PreCG.R, MFG.R, IFGoperc.R, ROL.R, and THA.L correlated significantly with the cognitive performances (indicated by executive function, processing speed, and episodic memory) in the ILA patients, suggesting that the abnormal nodal efficiencies of frontal–subcortical and limbic system areas might be involved in the psychopathology and pathophysiology of cognitive dysfunction in ILA. Some DTI studies also found that some projection fibers connecting thalamus to cortical regions (i.e., anterior thalamic radiation) are correlated with cognitive decline in SVD patients with high WMHs load (Duering et al., 2011, 2014). Specifically, a recent structural MRI study showed that increased nodal path length in the left inferior frontal gyrus (IFGoperc.L) acted as a mediator between periventricular WMHs (PWMH) and memory deficit (Yang et al., 2020). In addition, resting-state functional MRI studies based on graph theory analysis have shown that nodal global efficiency in frontal and parietal regions mediated the associations between processing speed and PWMH in WMH subjects (Chen et al., 2019). Thus, our findings provided further evidence that the WM network deficits in the frontal–subcortical and limbic system areas could explain the frequently occurring cognitive decrements (e.g., executive function and processing speed) in ILA.

Several limitations should be addressed. First, our study is limited by its cross-sectional design, which implies that no causal inferences about temporality of alterations in ILA can be made; thus, longitudinal and large-sample studies are warranted to further validate the effect of vascular lesions on the conversion of ILA to vascular dementia and to evaluate clinical values of WM and network measures to predict longitudinal changes. Second, further studies including ILA patients with and without cognitive impairment are important to investigate whether connectome-based measures could serve as a useful disease marker to early identify and monitor the disease and study therapeutic interventions in ILA. Third, the present two groups were not well matched for age, although the age effect was removed in all of the neuropsychological and network analyses. Therefore, these data should be interpreted cautiously. Fourth, we selected a widely used atlas, AAL-90, to define network nodes (Lawrence et al., 2014; Yang et al., 2020). However, it is a relative labeling technique that comprises differently sized brain regions that may influence the network properties (Zalesky et al., 2010). Using alternative techniques such as functional activations or high-resolution random parcellation to define nodes may provide a more interpretable solution (Fornito et al., 2013; Vergoossen et al., 2021). Furthermore, in this study, we focused on the WM changes in ILA patients. However, the spatial relationships between brain functional connectivity and structural changes require further exploration in our future research. Finally, further studies employing more advanced imaging methodologies to map the brain connectome and segment lesions appropriately and precisely may provide more comprehensive insights into the connectome-based mechanisms underlying ILA-related cognitive impairment (Ribaldi et al., 2021).

## Conclusion

In conclusion, we demonstrated that there are widespread WM integrity and structural network disruptions in ILA, and these disturbances are related to the severity of cognitive impairment. Our study enhances our understanding of the neuropathological process in ILA and subsequent cognitive impairment in subjects with high WMHs load.

## Data Availability Statement

The original contributions presented in the study are included in the article/Supplementary Material, further inquiries can be directed to the corresponding author.

## Ethics Statement

The studies involving human participants were reviewed and approved by the Research Ethics Committee of Zhongda Hospital Affiliated to Southeast University. The patients/participants provided their written informed consent to participate in this study. Written informed consent was obtained from the individual(s) for the publication of any potentially identifiable images or data included in this article.

## Author Contributions

All authors listed have made a substantial, direct and intellectual contribution to the work, and approved it for publication.

## Conflict of Interest

The authors declare that the research was conducted in the absence of any commercial or financial relationships that could be construed as a potential conflict of interest.

## Footnotes

1 http://www.fmrib.ox.ac.uk/fsl/

2 http://www.nitrc.org/projects/panda/
