# Supplementary material for: Disrupted Structural Brain Connectome Is Related to Cognitive Impairment in Patients With Ischemic Leukoaraiosis
Source: Front Hum Neurosci. 2021 Jun 10;15:654750. doi: 10.3389/fnhum.2021.654750 (PMC8223255; doi:10.3389/fnhum.2021.654750)
Supplement: Supplementary file 1 [file Table_1.DOC]

***Supplementary Material***

**1 Supplementary Methods**

**Participants**

All ILA patients had to satisfy the following inclusion criteria: (1) diffuse or confluent hyperintensity lesions (Fazekas rating score ≥ 3) in the subcortical or periventricular WM on T_2_-weighted or FLAIR images; (2) cortical or subcortical infarcts (diameter ≤ 15 mm); (3) absence of other neurological disease, including multiple sclerosis, Alzheimer’s disease, Parkinson’s disease, epilepsy, or head trauma; (4) absence of severe internal diseases, including heart diseases, renal failure diseases, liver diseases, tumor, or other systemic diseases; (5) absence of neuropsychological disorders or mental disease; (6) WM lesions unrelated to vascular diseases (e.g., immune, demyelination, metabolism, toxicity, infection, and other factors; and (7) no MRI contraindications. Notably, participants with structural abnormalities on MRI and those with recent (e.g., < 3 months before inclusion) ischemic or hemorrhagic stroke were excluded.

**Network Construction**

The procedure used to define the network nodes has been previously detailed (Gong et al., 2009; Shu et al., 2011) and was performed in this study using SPM8 software (http://www.fil.ion.ucl.ac.uk/spm/software/spm8). In brief, individual T1-weighted images were coregistered to the b0 images in the DTI space. The transformed T1 images were then non-linearly transformed into the ICBM 152 T1 template in the MNI space. The inverse transformations were used to warp the automated anatomical labeling (AAL) template (Tzourio-Mazoyer et al., 2002) from the MNI space to the DTI native space. To be noted, discrete labeling values were preserved using a nearest-neighbor interpolation method. Using this procedure, we obtained 90 cortical and subcortical regions (45 for each hemisphere, **Supplementary Table S2**), each representing a node of the WM network.

To define the network edges of the 90 regions, we selected a threshold value for the fiber bundles. Two regions were considered structurally connected if at least three fibers (*T* = 3) with two endpoints were located in these two regions (Bai et al., 2012). Such a threshold selection reduced the false-positive connectivity that resulted from noise or the limitations in the deterministic tractography and simultaneously ensured the size of the largest connected component in the networks across all of the samples. After defining the network edges, the weighted network analyses were performed. Specifically, we defined the fiber number (FN) of the connected fibers between two regions as the weights of the network edges. As a result, we constructed the FN-weighted WM network for each subject, which was represented by a symmetric 90 × 90 connectivity matrix.

**Statistical Analysis**

**Network Topological Metrics**

Group differences in topological properties were explored by the nonparametric permutation tests (Bullmore et al., 1999). Briefly, we first calculated the between-group difference in the mean value of each network metric. To test the null hypothesis that the observed group differences could occur by chance, we randomly reallocated each subject to one of the two groups and recomputed the mean differences between the two randomized groups. The randomization procedure was repeated 10,000 times, and a randomized null distribution based on between-group differences in each metric was created. Then the 95% percentile point of the distribution was used as the critical value for two-tail test of the null hypothesis. Notably, before the permutation tests, multiple linear regression analyses were applied to remove the confounding effects of age, sex, and education for each network metric.

Permutation tests were used to determine the significant levels of altered connectivity networks in the network-based statistic (NBS) analysis. Briefly, we first detected the significant nonzero connections within each group by performing multiple one-sample *t*-tests. Next, the nonzero connections within either the patient or control groups were combined into a connection mask. The NBS approach was conducted within the connection mask, where a primary threshold (*P* = 0.05) was first applied to a *t*-statistic (two-sample one-tailed *t*-tests). This *t*-statistic was performed for each link to define a set of suprathreshold links among which any connected components and their size (defined as the number of links included in these components) were determined. To estimate the significance of each component, the null distribution of the connected component size was empirically derived using a nonparametric permutation approach (5,000 permutations). For each permutation, all subjects were reallocated randomly into two groups, and the *t* statistic was conducted independently for each link. Subsequently, the same threshold (i.e., *P* = 0.05) was used to generate suprathreshold links among which the maximal connected component size was recorded. Finally, for a connected component of size M found in the right grouping of controls and patients, the corrected *P*-value was determined by calculating the proportion of the 5,000 permutations for which the maximal connected component was larger than M. Of note, the effects of age, sex, and education level were removed for each network metric by a regression analysis prior to the permutation tests.”

**2 Supplementary Tables**

**2.1 Supplementary Table S1.** The 50 WM tract ROIs based on the ICBM-DTI-81 WM labels atlas within cerebral regions

| Index | Abbr. | Tracts |
| --- | --- | --- |
| 1 | MCP | Middle cerebellar peduncle |
| 2 | PCT | Pontine crossing tract (a part of MCP) |
| 3 | gCC | Genu of corpus callosum |
| 4 | bCC | Body of corpus callosum |
| 5 | sCC | Splenium of corpus callosum |
| 6 | cbFN | Fornix (column and body of fornix) |
| 7 | CT.R | Corticospinal tract.R |
| 8 | CT.L | Corticospinal tract.L |
| 9 | ML.R | Medial lemniscus.R |
| 10 | ML.L | Medial lemniscus.L |
| 11 | ICP.R | Inferior cerebellar peduncle.R |
| 12 | ICP.L | Inferior cerebellar peduncle.L |
| 13 | SCP.R | Superior cerebellar peduncle.R |
| 14 | SCP.L | Superior cerebellar peduncle.L |
| 15 | CP.R | Cerebral peduncle.R |
| 16 | CP.L | Cerebral peduncle.L |
| 17 | ALIC.R | Anterior limb of internal capsule.R |
| 18 | ALIC.L | Anterior limb of internal capsule.L |
| 19 | PLIC.R | Posterior limb of internal capsule.R |
| 20 | PLIC.L | Posterior limb of internal capsule.L |
| 21 | RIC.R | Retrolenticular part of internal capsule.R |
| 22 | RIC.L | Retrolenticular part of internal capsule.L |
| 23 | ACR.R | Anterior corona radiata.R |
| 24 | ACR.L | Anterior corona radiata.L |
| 25 | SCR.R | Superior corona radiata.R |
| 26 | SCR.L | Superior corona radiata.L |
| 27 | PCR.R | Posterior corona radiata.R |
| 28 | PCR.L | Posterior corona radiata.L |
| 29 | PTR.R | Posterior thalamic radiation (include optic radiation).R |
| 30 | PTR.L | Posterior thalamic radiation (include optic radiation).L |
| 31 | SS.R | Sagittal stratum (include inferior longitidinal fasciculus and inferior fronto-occipital fasciculus).R |
| 32 | SS.L | Sagittal stratum (include inferior longitidinal fasciculus and inferior fronto-occipital fasciculus).L |
| 33 | EC.R | External capsule.R |
| 34 | EC.L | External capsule.L |
| 35 | CCG.R | Cingulum (cingulate gyrus).R |
| 36 | CCG.L | Cingulum (cingulate gyrus).L |
| 37 | CH.R | Cingulum (hippocampus).R |
| 38 | CH.L | Cingulum (hippocampus).L |
| 39 | F/ST.R | Fornix (cres) / Stria terminalis (can not be resolved with current resolution).R |
| 40 | F/ST.L | Fornix (cres) / Stria terminalis (can not be resolved with current resolution).L |
| 41 | SLF.R | Superior longitudinal fasciculus.R |
| 42 | SLF.L | Superior longitudinal fasciculus.L |
| 43 | SFOF.R | Superior fronto-occipital fasciculus (could be a part of anterior internal capsule).R |
| 44 | SFOF.L | Superior fronto-occipital fasciculus (could be a part of anterior internal capsule).L |
| 45 | IFOF.R | Inferior fronto-occipital fasciculus.R |
| 46 | IFOF.L | Inferior fronto-occipital fasciculus.L |
| 47 | UF.R | Uncinate fasciculus.R |
| 48 | UF.L | Uncinate fasciculus.L |
| 49 | TAP.R | Tapetum.R |
| 50 | TAP.L | Tapetum.L |

**2.2 Supplementary Table S2.** Cortical and sub-cortical regions defined in automated anatomical labeling template image

| Index | Brain regions | Abbr. | Index | Brain regions | Abbr. | Index | Brain regions | Abbr. |
| --- | --- | --- | --- | --- | --- | --- | --- | --- |
| 1 | Precentral_L | PreCG.L | 31 | Cingulum_Ant_L | ACG.L | 61 | Parietal_Inf_L | IPL.L |
| 2 | Precentral_R | PreCG.R | 32 | Cingulum_Ant_R | ACG.R | 62 | Parietal_Inf_R | IPL.R |
| 3 | Frontal_Sup_L | SFGdor.L | 33 | Cingulum_Mid_L | DCG.L | 63 | SupraMarginal_L | SMG.L |
| 4 | Frontal_Sup_R | SFGdor.R | 34 | Cingulum_Mid_R | DCG.R | 64 | SupraMarginal_R | SMG.R |
| 5 | Frontal_Sup_Orb_L | ORBsup.L | 35 | Cingulum_Post_L | PCG.L | 65 | Angular_L | ANG.L |
| 6 | Frontal_Sup_Orb_R | ORBsup.R | 36 | Cingulum_Post_R | PCG.R | 66 | Angular_R | ANG.R |
| 7 | Frontal_Mid_L | MFG.L | 37 | Hippocampus_L | HIP.L | 67 | Precuneus_L | PCUN.L |
| 8 | Frontal_Mid_R | MFG.R | 38 | Hippocampus_R | HIP.R | 68 | Precuneus_R | PCUN.R |
| 9 | Frontal_Mid_Orb_L | ORBmid.L | 39 | ParaHippocampal_L | PHG.L | 69 | Paracentral_Lobule_L | PCL.L |
| 10 | Frontal_Mid_Orb_R | ORBmid.R | 40 | ParaHippocampal_R | PHG.R | 70 | Paracentral_Lobule_R | PCL.R |
| 11 | Frontal_Inf_Oper_L | IFGoperc.L | 41 | Amygdala_L | AMYG.L | 71 | Caudate_L | CAU.L |
| 12 | Frontal_Inf_Oper_R | IFGoperc.R | 42 | Amygdala_R | AMYG.R | 72 | Caudate_R | CAU.R |
| 13 | Frontal_Inf_Tri_L | IFGtriang.L | 43 | Calcarine_L | CAL.L | 73 | Putamen_L | PUT.L |
| 14 | Frontal_Inf_Tri_R | IFGtriang.R | 44 | Calcarine_R | CAL.R | 74 | Putamen_R | PUT.R |
| 15 | Frontal_Inf_Orb_L | ORBinf.L | 45 | Cuneus_L | CUN.L | 75 | Pallidum_L | PAL.L |
| 16 | Frontal_Inf_Orb_R | ORBinf.R | 46 | Cuneus_R | CUN.R | 76 | Pallidum_R | PAL.R |
| 17 | Rolandic_Oper_L | ROL.L | 47 | Lingual_L | LING.L | 77 | Thalamus_L | THA.L |
| 18 | Rolandic_Oper_R | ROL.R | 48 | Lingual_R | LING.R | 78 | Thalamus_R | THA.R |
| 19 | Supp_Motor_Area_L | SMA.L | 49 | Occipital_Sup_L | SOG.L | 79 | Heschl_L | HES.L |
| 20 | Supp_Motor_Area_R | SMA.R | 50 | Occipital_Sup_R | SOG.R | 80 | Heschl_R | HES.R |
| 21 | Olfactory_L | OLF.L | 51 | Occipital_Mid_L | MOG.L | 81 | Temporal_Sup_L | STG.L |
| 22 | Olfactory_R | OLF.R | 52 | Occipital_Mid_R | MOG.R | 82 | Temporal_Sup_R | STG.R |
| 23 | Frontal_Sup_Medial_L | SFGmed.L | 53 | Occipital_Inf_L | IOG.L | 83 | Temporal_Pole_Sup_L | TPOsup.L |
| 24 | Frontal_Sup_Medial_R | SFGmed.R | 54 | Occipital_Inf_R | IOG.R | 84 | Temporal_Pole_Sup_R | TPOsup.R |
| 25 | Frontal_Med_Orb_L | ORBsupmed.L | 55 | Fusiform_L | FFG.L | 85 | Temporal_Mid_L | MTG.L |
| 26 | Frontal_Med_Orb_R | ORBsupmed.R | 56 | Fusiform_R | FFG.R | 86 | Temporal_Mid_R | MTG.R |
| 27 | Rectus_L | REC.L | 57 | Postcentral_L | PoCG.L | 87 | Temporal_Pole_Mid_L | TPOmid.L |
| 28 | Rectus_R | REC.R | 58 | Postcentral_R | PoCG.R | 88 | Temporal_Pole_Mid_R | TPOmid.R |
| 29 | Insula_L | INS.L | 59 | Parietal_Sup_L | SPG.L | 89 | Temporal_Inf_L | ITG.L |
| 30 | Insula_R | INS.R | 60 | Parietal_Sup_R | SPG.R | 90 | Temporal_Inf_R | ITG.R |

**2.3 Supplementary Table S3. Comparisons of the global network measures between the ILA and HC groups**

|  | **HCs**  (n =87) | **ILA**  (n = 87) | ***P* value** |
| --- | --- | --- | --- |
| Clustering coefficient (*Cp*) | 0.38 ± 0.03 | 0.37 ± 0.03 | 0.139 |
| Characteristic path length (*Lp*) | 1.33 ± 0.22 | 1.52 ± 0.34 | < 0.001 |
| Gamma (*γ*) | 6.83 ± 0.83 | 7.29 ± 1.11 | 0.001 |
| Lambda (*λ*) | 1.37 ± 0.09 | 1.43 ± 0.14 | 0.139 |
| Sigma (*σ*) | 5.00 ± 0.58 | 5.11 ± 0.74 | 0.135 |
| Local efficiency (*E_loc_*) | 1.71 ± 0.33 | 1.64 ± 0.40 | 0.092 |
| Global efficiency (*E_glob_*) | 0.77 ± 0.12 | 0.69 ± 0.16 | < 0.001 |

Data are presented as the mean ± standard deviation.

Abbreviations: ILA, ischemic leukoaraiosis; HCs, healthy controls.

**3** **Supplementary Results**

**3.1 Connectivity-based Analysis**

We utilized the NBS method to identify a single connected component with 4 nodes and 3 connections, which was universally decreased in the ILA patients (*P* = 0.042, corrected) (Figure S1). The involved nodal regions mainly included the right SFGmed, right ORBsupmed, right ORBsup, and right CAU.”


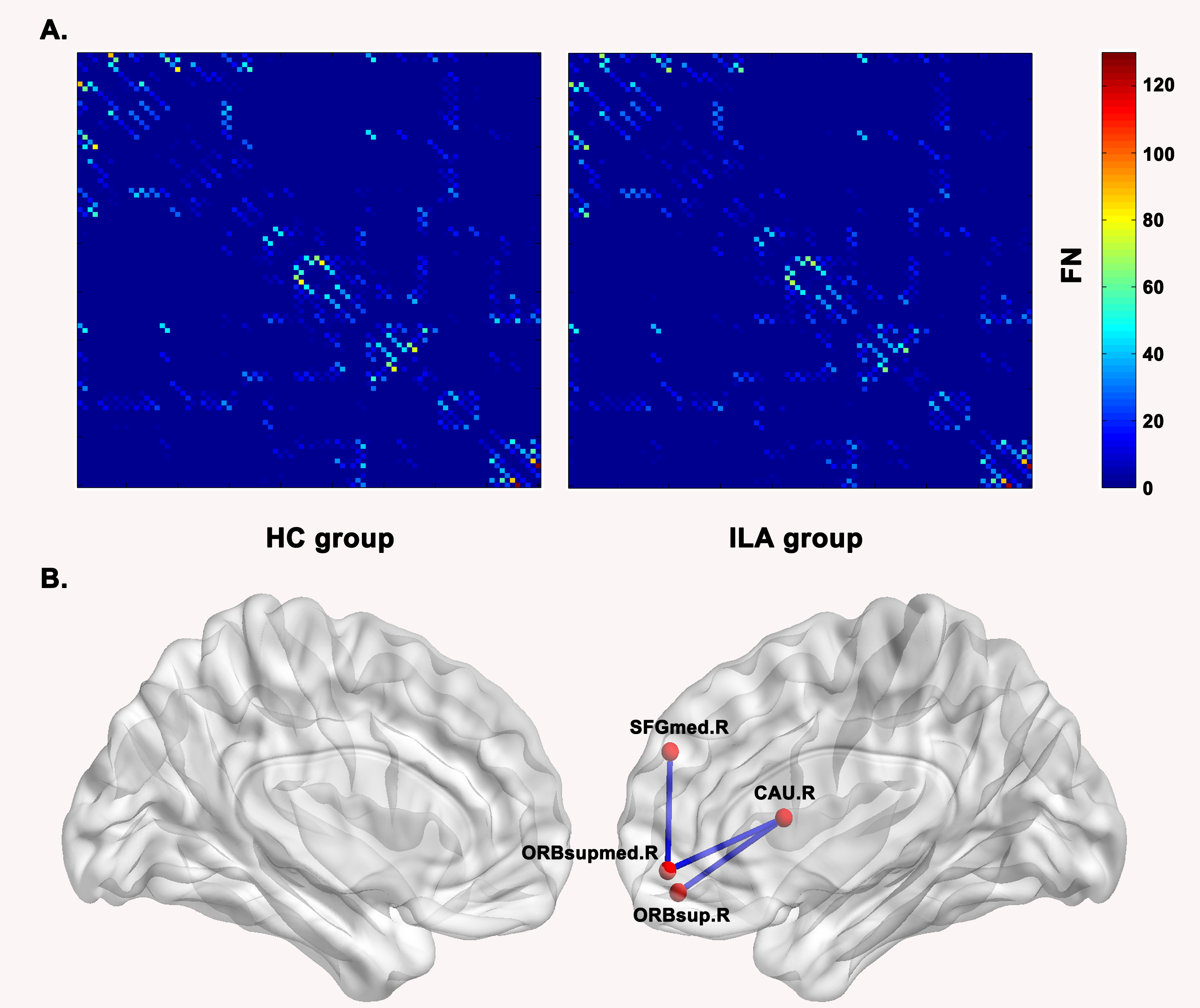


**Figure S1. Decreased connectivity of the white matter structural network in patients with ILA relative to controls.** **(A)** The mean FN matrices for both the HC and ILA groups. **(B)** The connected network showing decreased structural connections in the ILA patients compared with healthy controls. For the abbreviations of nodes, see Supplementary Table S2. ILA, ischemic leukoaraiosis; HC, healthy controls; FN, fiber number. L, left; R, right.

**3.2 Supplementary Figure S2**


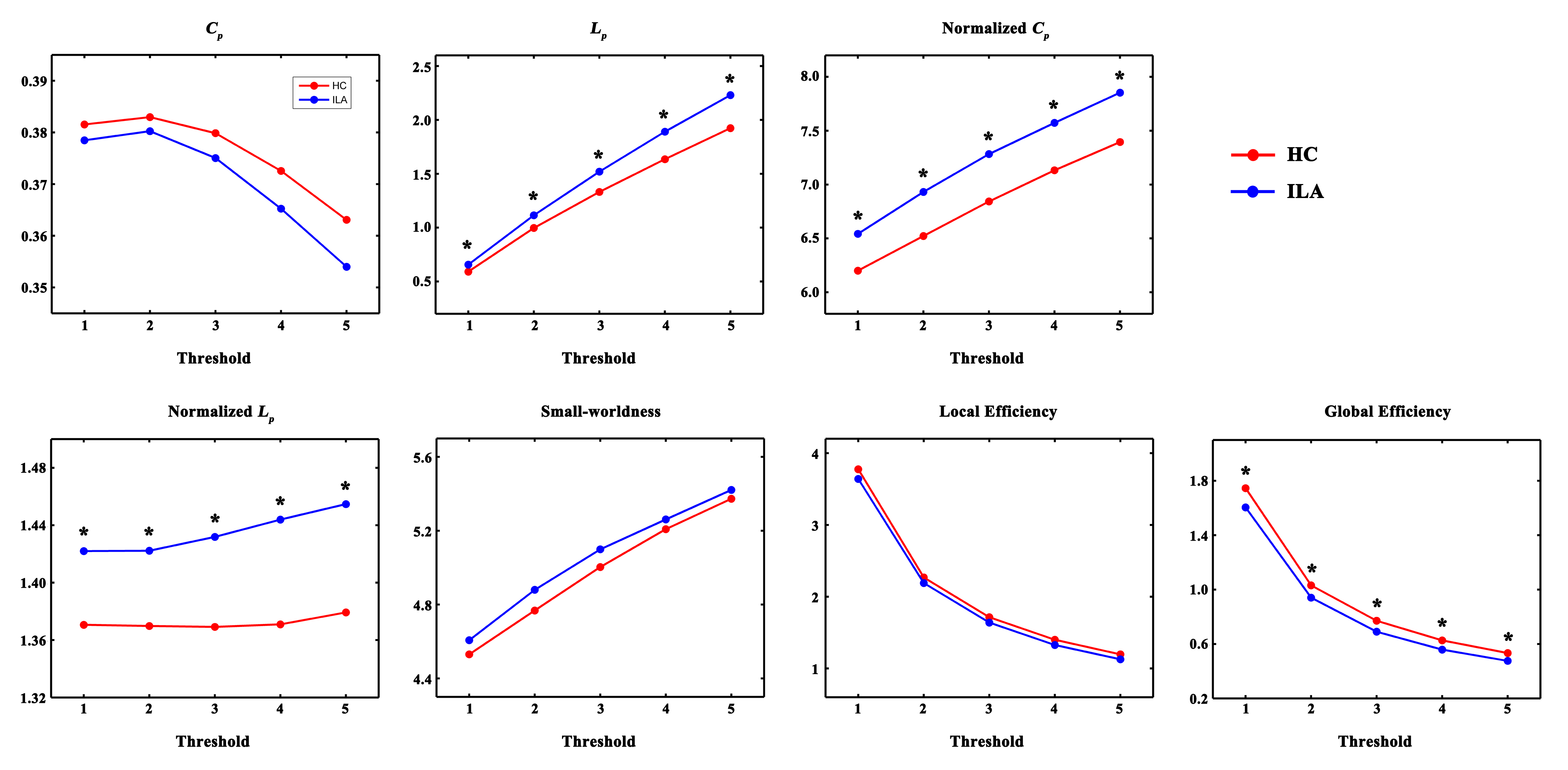


**Figure S2. Global network measures of WM structural networks were quantified in the HC and ILA patients.** The black asterisks (*) represent significant between-group differences (*P* < 0.05) between the ILA (blue line) and HC (red line) groups. ILA, ischemic leukoaraiosis; HC, healthy controls.

**Supplemental Reference**

Bai, F., Shu, N., Yuan, Y., Shi, Y., Yu, H., Wu, D., et al. (2012). Topologically convergent and divergent structural connectivity patterns between patients with remitted geriatric depression and amnestic mild cognitive impairment. Journal of Neuroscience 32, 4307-4318. doi: 10.1523/JNEUROSCI.5061-11.2012

Bullmore E. T., Suckling J., Overmeyer S., Rabe-Hesketh S., Taylor E., and Brammer M. J. (1999). Global, voxel, and cluster tests, by theory and permutation, for a difference between two groups of structural MR images of the brain. IEEE Trans Med Imaging 18, 32-42. doi: 10.1109/42.750253

Gong, G., He, Y., Concha, L., Lebel, C., Gross, D. W., Evans, A. C., et al. (2009). Mapping anatomical connectivity patterns of human cerebral cortex using in vivo diffusion tensor imaging tractography. Cerebral Cortex 19, 524-536. doi: 10.1093/cercor/bhn102

Shu, N., Liu, Y., Li, K., Duan, Y., Wang, J., Yu, C., et al. (2011). Diffusion tensor tractography reveals disrupted topological efficiency in white matter structural networks in multiple sclerosis. Cerebral Cortex 21, 2565-2577. doi: 10.1093/cercor/bhr039

Tzourio-Mazoyer, N., Landeau, B., Papathanassiou, D., Crivello, F., Etard, O., Delcroix, N., et al. (2002). Automated anatomical labeling of activations in SPM using a macroscopic anatomical parcellation of the MNI MRI single-subject brain. NeuroImage 15, 273-289. doi: 10.1006/nimg.2001.0978
